# Regulation of CO_2_ Concentrating Mechanism in Cyanobacteria

**DOI:** 10.3390/life5010348

**Published:** 2015-01-28

**Authors:** Robert L. Burnap, Martin Hagemann, Aaron Kaplan

**Affiliations:** 1 Department of Microbiology and Molecular Genetics, Henry Bellmon Research Center, Oklahoma State University, Stillwater, OK 74078, USA; 2 Institute Biosciences, Department Plant Physiology, University of Rostock, Albert-Einstein-Straße 3, Rostock D-18059, Germany; E-Mail: martin.hagemann@uni-rostock.de; 3 Department of Plant and Environmental Sciences, The Alexander Silberman Institute of Life Sciences, Edmond J. Safra Campus, Givat Ram, Hebrew University of Jerusalem, Jerusalem 91904, Israel; E-Mail: aaron.kaplan@mail.huji.ac.il

**Keywords:** CO_2_-concentrating mechanism, metabolic signals, non-coding RNA, transcription factor, photosynthesis, RubisCO

## Abstract

In this chapter, we mainly focus on the acclimation of cyanobacteria to the changing ambient CO_2_ and discuss mechanisms of inorganic carbon (C_i_) uptake, photorespiration, and the regulation among the metabolic fluxes involved in photoautotrophic, photomixotrophic and heterotrophic growth. The structural components for several of the transport and uptake mechanisms are described and the progress towards elucidating their regulation is discussed in the context of studies, which have documented metabolomic changes in response to changes in C_i_ availability. Genes for several of the transport and uptake mechanisms are regulated by transcriptional regulators that are in the LysR-transcriptional regulator family and are known to act in concert with small molecule effectors, which appear to be well-known metabolites. Signals that trigger changes in gene expression and enzyme activity correspond to specific “regulatory metabolites” whose concentrations depend on the ambient C_i_ availability. Finally, emerging evidence for an additional layer of regulatory complexity involving small non-coding RNAs is discussed.

## 1. General Description of Function and Components of the Cyanobacterial CCM

Photosynthetic microorganisms including cyanobacteria are capable of acclimating and growing under a wide range of ambient CO_2_ concentrations. The process of acclimation is mediated via a syndrome of changes, at various cellular levels, including modulation of the expression of genes involved in the operation of the CO_2_ concentrating mechanism (CCM) [1,2,3,4,5,6,7,8]. The existence of a CCM was first recognized in the green alga *Chlamydomonas reinhardtii*, [9] and the cyanobacterium *Anabaena variabilis* [10]. Studies on the CCM initially focused on the physiological/biochemical aspects. Isolation of mutants impaired in various aspects of its activity and the development of molecular tools led to an emphasis upon the genetic/molecular aspects. The CCM enables photosynthetic microorganisms to raise the CO_2_ level at the carboxylating sites, carboxysomes in prokaryotes and pyrenoids in eukaryotes, and thereby overcome the large difference (approximately 5–20-fold, in green algae and cyanobacteria, respectively) between the K_m_(CO_2_) of their carboxylating enzyme ribulose 1,5-bisphosphate carboxylase/oxygenase (RubisCO) and the concentration of dissolved CO_2_ at equilibrium with air. The efficiency of the CCM may be deduced from the ratio between the apparent whole cell photosynthetic affinity for extracellular CO_2_ and the enzymatic affinity, K_m_(CO_2_) of RubisCO; values as high as 1000 can be observed in cyanobacteria, particularly at alkaline environments, where the amount of free CO_2_ is very low and the cells are mainly consuming bicarbonate from the medium.

Light energy is being used to fuel the accumulation of inorganic carbon (C_i_) within the cells and to maintain the cytoplasmic CO_2_ concentration much lower than expected at chemical equilibrium; thereby, providing the gradient for inward diffusion of CO_2_ and minimizes its leak from the cells. In addition to compensating for the relatively low affinity of RubisCO for CO_2_, the elevation of CO_2_ concentration at the carboxylating site activates the enzyme [11] and depresses photorespiration [12,13]. The very large transmembrane Ci fluxes involved in the operation of the CCM—as much as 8–10 fold higher than the photosynthetic rate [14]—may help to dissipate excess light energy and impose a significant load on the pH homeostasis of the cells. In fact, a mutant of *Synechocystis* sp. PCC 6803 where all the five known components involved in bicarbonate uptake and internal conversion of CO_2_ to HCO_3_^−^ is able to grow under a high level of CO_2_ (HC, 1%–8% CO_2_ in air) but undergo photodamage when exposed to an elevated illumination [15].

**Figure 1:**
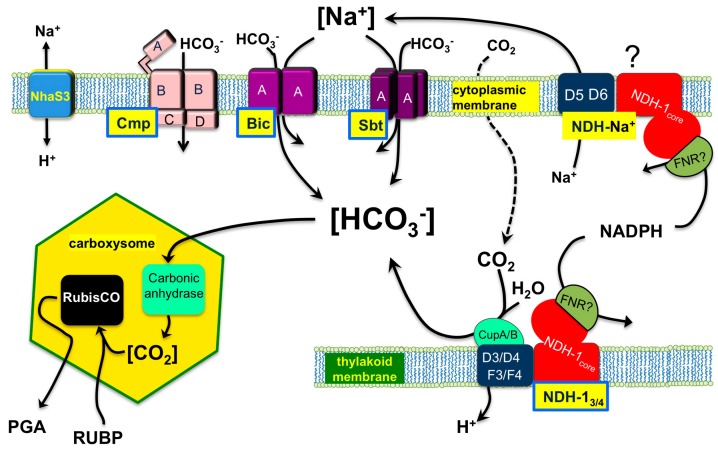
Schematic representation of the cyanobacterial CO_2_ concentrating mechanism (CCM).

Many constituents are involved in the operation of the cyanobacterial CCM. Generally speaking, these components may be grouped according to those involved in the intracellular accumulation of C_i_, including the entities engaged in CO_2_ uptake and bicarbonate transport, and those taking part in CO_2_ elevation and consumption within the carboxysomes (Figure 1).

CO_2_ that crosses the cell envelopes by diffusion via the aquaporins [16,17,18] or generated from the HCO_3_^−^ in the carboxysomes and the cytoplasmic pool is converted to HCO_3_^−^ by the so-called CO_2_ uptake systems that involve thylakoid membrane-located NDH-1 complexes [1,19,20,21,22,23,24].

This is a process that uses cellular energy and, therefore, the CO_2_ hydration reaction is driven far towards the HCO_3_^−^ product. Because these systems are not transporting CO_2_, but consuming it with high efficiency into HCO_3_^−^, they maintain a diffusion gradient to facilitate rapid net CO_2_ flux into the cell. Two CO_2_ uptake systems were recognized in *Synechocystis* sp. PCC 6803, often used as a model cyanobacterium. The high affinity, Ndh-1_3_, is strongly upregulated when the cells are exposed to a limiting CO_2_ level. The subunits are encoded by *ndhF3*, *ndhD3*, *cupA* and *sll1735*. The low affinity Ndh-1_4_ system is constitutively transcribed and encoded by *ndhF4*, *ndhD4*, *cupB* [23,25,26,27,28]. The central membrane component of the respiratory Ndh-1 complex, NdhB, is involved in both systems; its inactivation results in a high CO_2_ requiring mutant unable to take up CO_2_ but also inability to utilize extracellular glucose because of impaired cyclic electron transport [27]. An additional, more poorly understood NDH-1 complex containing the NdhD5 and NdhD6 subunits has been tentatively assigned (see “?” in Figure 1) a function in augmenting the Na^+^ gradient across the cytoplasmic membrane based upon findings with *Anabaena* Na^+^ tolerance [29], sequence similarities to the *Bacillus* MRP system [30], and the fact that it is coordinately regulated with the Na^+^/HCO_3_^−^ symporter, SbtA, discussed below [31,32]. Despite impressive progress in the clarification of the Ndh-1 subunit composition and its organization [21,33], the mechanism of CO_2_ conversion to HCO_3_^−^ in the thylakoid membranes and its association with the photosynthetic electron transport is not understood [2,26]. Clearly, the HCO_3_^−^ produced is released into an environment where its concentration may exceed 50 mM,* i.e.*, against the chemical equilibrium.

The two CO_2_ uptake systems are present in the ancestral cyanobacterium *Gloeobacter violaceus* suggesting that they might have been acquired at an early stage of cyanobacterial evolution before the branching of α-cyanobacterial lineage. The genes essential for CO_2_ uptake are missing in *Prochlorococcus* strains. Moreover, the low-CO_2_-inducible system, Ndh-1_3_, is absent in some of the marine picoplanktonic *Synechococcus* strains belonging to the α-cyanobacteria, but it is present in the β-cyanobacterium *Synechococcus* strain PCC 7002. Thus, there is more room for future phylogenetic analysis in an attempt to clarify the development of the CO_2_ uptake systems in cyanobacteria. Indeed, with the increasing number of cyanobacterial genomes now available [34], it may soon be possible to better resolve this question by the application of a more comprehensive phylogenetic analysis of the genes encoding the CCM.

Three types of HCO_3_^−^ transporter, located in the cytoplasmic membrane, have been identified, mostly through studies of *Synechococcus* *elongatus* sp. PCC 7942, *Synechocystis* sp. PCC 6803 and *Synechococcus* sp. PCC 7002 [1,22,26]. The first was BCT1, an ATP-binding cassette (ABC)-type high affinity HCO_3_^−^ transporter encoded by *cmpA-D* [35]. The reason for the dependence of cyanobacterial photosynthesis and growth in the presence of a few mM Na^+^ ions (see [2] and references therein) became clear when the Na^+^/HCO_3_^−^ symporter, SbtA, a high affinity sodium-dependent HCO_3_^−^ transporter was recognized [36]. Finally, BicA, a SulP-type low affinity, high V_max_ sodium dependent HCO_3_^−^ transporter was described [37]. Driving of HCO_3_ uptake against its electrochemical gradient likely occurs at the expense of Na^+^ ions. Regulation of the Na^+^/HCO_3_^−^ symporters shows considerable diversity: In *Synechocystis* sp. PCC 6803, BicA appears to be constitutively transcribed, whereas both SbtA and BCT1 are transcriptionally upregulated when the cells are exposed to limiting CO_2_ levels [25,32]. On the other hand, both SbtA and BicA are upregulated at the transcriptional level in *Synechococcus* sp. PCC 7002 [38]. Additionally, *sbtA* is the first gene in what often appears to be a dicistronic operon with the second gene, tentatively designated *sbtB*, encoding a protein assigned to a periplasmic location [39].

Significant species-specific differences were reported with respect to the nature of the C_i_ species taken up from the medium by various cyanobacteria [40]. Constitutive presence of high sodium ion levels in the marine environment but low and fluctuating sodium amounts in the fresh waters may have contributed to the reliance of those inhabiting the latter on the ABC transporter rather than Na^+^-dependent mechanisms. The preferred C_i_ source is also strongly affected by the ambient conditions, particularly the pH. For example, *Microcystis* strains have been described that lost either the SbtA or the BicA bicarbonate transport system and showed corresponding growth differences at low or high inorganic carbon levels in the environment [40]. Most of the α-cyanobacteria seem to miss the well-characterized bicarbonate transporters found in β-cyanobacteria. However, a recent bioinformatics survey identified candidate proteins in α-cyanobacteria, which show some similarity to established bicarbonate transporters among cyanobacteria or even might represent novel types of such transporters [41].

Regardless of the C_i_ species taken up, high amounts of HCO_3_^−^ accumulate in the cytoplasm and then penetrate into the carboxysomes, where it is converted to CO_2_, catalyzed by carbonic anhydrase (CA) in close proximity to RubisCO [2,42,43]. Two carboxysome types covering two subtypes of RubisCO have been found among cyanobacteria. The majority of cyanobacteria carry the β-type of carboxysomes and form 1B of RubisCO, whereas picoplanktonic cyanobacteria, mostly *Prochlorococcus*/*Synechococcus* spp., harbor α-carboxysomes and RubisCO form 1A [44,45]. The latter group is believed to have acquired the different carboxysome and RubisCO types via a lateral gene transfer event, based upon similarities with carboxysomes from other bacteria [46]. Among β-cyanobacteria, at least two CA types were found inside the carboxysome. Many strains carry genes for the β-type CA coded by *cca* [47]. Interestingly, this type of CA is missing in some β-cyanobacteria implying that another CA replaced Cca. The structural carboxysome protein CcmM exhibits significant sequence similarities to γ-type CAs in its N-terminal domain. Recently it was shown that a truncated CcmM protein indeed formed an active CA, the activity of which is strongly affected by the redox status of the cells [48]. In addition to the CAs inside the carboxysomes, there are hints of CA activity associated with the periplasm or outer surface of the cyanobacteria, possibly contributing to the cyanobacterial CCM [49].

Considerable progress was recently made in the elucidation of the structural organization of the carboxysomes and their function [7,40,43,50,51]. Bicarbonate that enters these microcompartments by diffusion is then converted to CO_2_, mediated by CA confined to these bodies, which is then consumed by RubisCO. All other enzymes of the Calvin-Benson-Bassham (CBB) cycle are located outside the carboxysomes. Thus, cyanobacterial photosynthesis involves a flux of ribulose 1,5-bisphosphate (RuBP) into and of 3-phosphoglycerate (3PGA) out of the carboxysomes. This diffusion is facilitated by pores that were found in the carboxysomal shell proteins CcmK and CcmO [43,46]. These proteins form hexamers and build the carboxysomal surface, while the pentameric CcmL is found at the edges. The inner architecture of the carboxysome is mostly determined by CcmM that is found in multiple forms [52]. In addition to its potential CA function, the CcmM is binding RubisCO to form a semicrystalline internal order. Recently, it has been shown that newly translated CcmM and RubisCO form defined aggregates that serve as nucleation cores for the synthesis of novel carboxysomes [50]. Further discussion of the carboxysomes structure and function is beyond the scope of this review.

**Figure 2:**
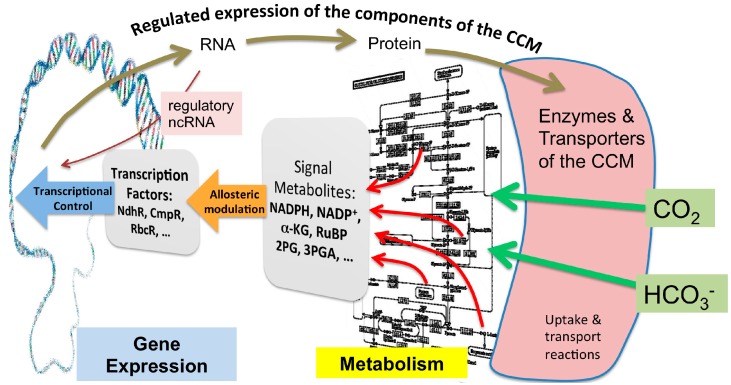
Overview of the different regulatory levels adjusting the activity of the CO_2_ concentrating mechanism (CCM) according to the ambient inorganic carbon levels.

The activity of the CCM is strongly affected by the concentration of CO_2_ experienced by the cells during growth. Indeed, the regulation of the CCM is a striking example of how cells may produce changes in physiological state in response to a single environmental parameter. Cyanobacteria grown under elevated CO_2_ concentrations exhibit a relatively lower apparent whole cell photosynthetic affinity for extracellular C_i_ (K_s_ ~200 μM) compared to cells adapted to low availability of CO_2_ (K_s_ ~10 μM) [10,53]. These are aggregate, whole cell affinities that reflect the changes in the abundance and kinetic characteristics of multiple transporters and CO_2_ uptake enzymes. Moreover, there is considerable phyletic variation in the actual composition and expression of these different C_i_-uptake “subsystems”, which is only gradually becoming apparent with advances in genomics [34]. Finally, the details of the regulatory mechanisms controlling the interchange between the low affinity state and the high affinity states are only beginning to emerge, but it is clear that regulation is exerted at multiple levels. As discussed below, regulation involves gene expression, with both transcriptional and post-transcriptional components, as well as modulation of the activity of the expressed transporters. Understanding the signals triggering these changes is also becoming better understood. It now appears that internal metabolic changes that occur in response to changing C_i_ availability are at the heart of this regulation. Accordingly, the signals that trigger changes in gene expression and enzyme activity correspond to specific “regulatory metabolites” whose concentrations predictably depend upon the ambient C_i_ availability (Figure 2). Therefore, an understanding of the regulation of the CCM appears to require both the detailed information about metabolic fluctuations, on the one hand, and the allosteric interactions between regulatory proteins and their cognate metabolic effector molecules, on the other.

## 2. Metabolomic Investigations of Carbon Metabolism and How It Pertains to the C_i_ Acquisition Mechanisms

The primary aim of the CCM is to saturate the main carboxylating enzyme RubisCO with CO_2_ inside the carboxysome. Labeling experiments using ^14^C showed that in addition to RubisCO alternative carboxylation reactions such as the C4-like activity via PEP carboxylase occur in cyanobacterial cells and may contribute to the carbon assimilation [54]. Especially after feeding ^14^C-bicarbonate, comparable labeling rates of malate and 3PGA were observed in exponential growing cells, and the malate as well as aspartate labeling became dominant in stationary phase cells [55]. Thus, these pioneer experiments indicated that RubisCO is the dominating carboxylating enzyme but additional reactions seem to contribute significantly to the overall carbon assimilation, at least under certain conditions. Recent labeling experiments with the stable isotope ^13^C showed that the RubisCO-catalyzed CO_2_ fixation is by far the most important carboxylating reaction and other enzymes play rather minor roles [56,57]. The latter study by Young* et al.* [57] combined labeling and a model approach,* i.e.*, they used the isotopically non-stationary metabolic flux analysis (INST-MFA). The INST-MFA approach allowed deducing carbon fluxes and showed that carbon fixation via PEP carboxylase is of low proportion. Moreover, this additional fixed carbon is mostly shuttled from malate to pyruvate to fill up the pyruvate pool for amino acid synthesis, whereby the malic enzyme is even releasing the freshly fixed carbon as CO_2_. As discussed in Section 3, it has become increasingly clear that the regulation of the CCM involves sensing the concentrations of several different metabolites that change in abundance in response to the inorganic carbon status of cells and thereby provide specific metabolic cues for the control of gene expression (Figure 2).

The labeling approaches mentioned above also revealed the major pathways for the utilization of the newly fixed carbon. In addition to the operation of the main pathways of the CBB, it was observed that photorespiratory flux is substantial in wild-type cells of *Synechocystis* sp. PCC 6803 despite the provision of ample CO_2_ by bubbling (air enriched with 5% CO_2_, HC). Photorespiration is a process that is essentially linked to oxygenic photosynthesis, because it metabolizes the toxic byproduct 2-phosphoglycolate (2PG) of the RubisCO oxygenase reaction [58]. Initially, it was thought that cyanobacteria are not performing photorespiration due to the CCM activity. However, during the last few years, it was shown that *Synechocystis* sp. PCC 6803 has not only the plant-like photorespiratory cycle to metabolize 2PG, but it also performs two other routes for the turnover of 2PG [12]. The measured photorespiratory flux in ^13^C-labeled *Synechocystis* cells was rather low; it amounted to between 0.5 and 1% of the carbon assimilation [59]. Nevertheless, this finding proved that despite the basal activity of the CCM and the CO_2_ supplement, RubisCO is performing the oxygenase reaction. This activity also indicates that at least a fraction of RubisCO is exposed to molecular oxygen and a reduced level of CO_2_. Whether this is due to the fact that the carboxysome is less tightly closed against O_2_ as previously assumed or that RubisCO is localized outside the carboxysome, cannot be decided in the moment. It has been shown that a substantial fraction of RubisCO seems to be located outside the carboxysome [60], which could be explained by the accumulation of newly translated RubisCO as the core for nascent carboxysomes [50]. As expected, the photorespiratory flux increased when cells of *Synechocystis* sp. PCC 6803 were shifted from HC into normal air (0.04% CO_2_, low carbon, LC). Especially in the early transition phase after the shift, when the CCM was not fully induced, the photorespiratory flux increased to 4% but returned to rather low levels after long-term LC exposure [56]. A much higher flux into photorespiratory 2PG metabolism was found modifying the carboxysome in the *ccmM* mutant of *Synechocystis* sp. PCC 6803 [61]. This experiment provided direct proof that CO_2_ enrichment and, perhaps also O_2_-shielding, inside the carboxysome are indeed responsible for the virtual absence of photorespiration in cyanobacterial wild-type cells. This also recalls the role of the rising CO_2_ concentration in the carboxysomes for the activation of RubisCO [11]. Nevertheless, even the low flux into 2-phosphoglycolate in wild-type cells needs to be recycled back to 3PGA or other useful metabolites, since model calculations predicted that growth will become more than 10% diminished if glycolate is excreted from LC grown cells instead of its recycling to useful intermediates [62]. This calculated growth deficit is also consistent with the finding that genes for the 2PG metabolism are found in all completely known cyanobacterial genomes [13].

Due to the CCM, the carbon flow into photorespiration is only slightly enhanced after shifts from HC into LC conditions. In addition to the photorespiratory flux, which is directly connected to the action and efficiency of the CCM, the cellular carbon partitioning becomes globally changed. These changes result in marked differences in steady state levels of certain metabolites, which are closely associated to the LC shift conditions and could potentially play a role as signal metabolites (e.g., αKG as discussed in detail below). Under HC, most of the newly fixed carbon is leaving the CBB cycle in the direction of the carbohydrate metabolism and storage, whereas under LC conditions, carbon is exported via glycolysis and the tricarboxylic acid (TCA) cycle to provide carbon skeletons for amino acid synthesis [56,63]. Thus, HC cells are characterized by a large pool of glycogen inside the cells [58], whereas LC cells in the presence of sufficient nitrogen sources are glycogen poor. It was found that HC cells have greater capacity to adapt to increased light intensity than LC cells in a way that suggests that they had greater metabolic reserves, likely glycogen, to mobilize an adaptive response [64]. The glycogen pool could be metabolized in the dark mainly via the oxidative pentose phosphate (OPP) cycle [65] to synthesize carbon skeletons, NADPH and ATP. Recently, it has been shown that the glycogen breakdown in the dark also is responsible for the refilling of CBB cycle intermediates to quickly restart carbon fixation in the light period [66]. However, even in cells growing in continuous light, the glycogen pool is obviously part of the cellular metabolism, since its content is kept at a steady state value beside the incorporation of newly fixed carbon. Moreover, the glycogen pool seems to play an essential role in buffering cellular metabolism against unbalanced N/C availability. Mutants of *Synechocystis* sp. PCC 6803 defective of glycogen accumulation showed an increased accumulation and release of pyruvate and α-ketoglutarate (αKG; a.k.a. 2-oxoglutarate, 2OG) under N-limiting conditions when grown under LC conditions [67]. An increased accumulation and excretion of soluble sugars was observed in HC-grown cells of a corresponding mutant of *Synechococcus* sp. PCC 7002 [68]. In addition to the changed metabolite fluxes, these mutants also showed changes in the cellular redox balance. Whether or not the glycogen metabolism also exerts a regulatory function or the observed effects are the consequence of the missing sink capacity still cannot be decided. Possibly, the altered accumulation of nucleotide sugars in these cells could play a role as metabolic signal, as has been shown for UDP-glucose in *E. coli* [69].

Despite the induction of the CCM, the transfer from HC to conditions of normal air (LC) results in carbon-limitation and significantly decreased growth rates (e.g., [58,60]). Probably, the growth decline is not only due to the lack of sufficient carbon but also results from the high energy demand of the CCM, since the induced C_i_ transporters are depending on cellular energy. Depending on the mechanism, it uses one ATP per bicarbonate (in the case of the BCT) or at least one proton per bicarbonate to export the co-transported Na^+^ back to the medium (in the cases of SbtA and BicA) (see Figure 1). The energetic cost of the CO_2_-uptake mechanism of the Ndh-1_3/4_ system is even less well understood, but it too is likely energetically expensive as reductant in the form of NADPH or ferredoxin is oxidized, probably to the level of plastoquinone, to drive the hydration of CO_2_ via the proton pumping mechanism [2,70]. As mentioned above, LC-grown cells use the remaining organic carbon mainly to support biosynthesis such as those of amino acids. They export carbon via glycolysis toward the TCA cycle that has been also demonstrated by the accumulation of 2PGA and PEP as well as some TCA cycle intermediates in LC-grown cyanobacterial cells [59,71]. Thus, the carbon is channeled in the direction of pyruvate and αKG, two important precursors of amino acid biosynthesis. The data regarding accumulation of αKG in LC-shifted cells were somehow contradictory, but an improved cultivation and sampling protocol revealed that αKG is accumulated in cells transferred from HC toward LC conditions [72] as has been shown before for N-limited cyanobacteria [73]. The increased flux to pyruvate and αKG became obvious, when those cells are transferred toward N-limiting conditions. In the absence of the sink amino acid biosynthesis, these compounds were released from the cells [67,74]. It is largely unknown how this redirection in carbon flow is regulated under changing carbon-conditions. Transcriptional analysis of LC-shifted cells of *Synechococcus elongatus* PCC 7942 revealed that not only genes coding for components of the CCM were transcriptionally activated but also genes for enzymes of the primary carbon metabolism [71]. However, the comparable metabolic changes in *Synechocystis* sp. PCC 6803 were not accompanied by marked changes in the transcription of genes for the corresponding enzymes. Thus, posttranscriptional mechanisms might be involved as has been also suggested for the increase of carboxysome numbers despite the rather weak transcriptional response of these genes [75]. It has been shown that many enzymes involved in primary carbon metabolism are modified via redox-sensitive cysteines [76,77] and/or phosphorylation [78]. Recently, it has been shown that the Ca^2+^-dependent phosphorylation of transketolase in chloroplasts of Arabidopsis exerts significant effects on the enzyme feature. The authors concluded that the phosphorylation of this enzyme participating in the CBB and OPP cycles can play a crucial role for the carbon allocation in the chloroplast [79].

An unexpected addition to the regulation of carbon fixation and possibly CCM was recently provided by the comparisons of C-fixation in the *Microcystis aeruginosa* PCC 7806 wild type and the mutant defective in the production of the toxin microcystin. It has been shown that microcystin can be bound to different target proteins including RubisCO and other enzymes of the CBB cycle in the wild type [80]. In the case of RubisCO, the microcystin binds to defined thiol groups and the binding pocket resembles the microcystin-binding toward certain eukaryotic protein phosphatases when acting as a toxin. These data and the lowered light-resistance of the mutant indicated that microcystin might play a role as regulator of cellular metabolism. This assumption was supported by recent experiments with high-light-stressed cells of *M. aeruginosa*. These cells showed an increased accumulation of photorespiratory intermediates such as glycolate [81] supporting the notion that photorespiration is one means to dissipate cellular energy under oxidative stress conditions to regenerate acceptors for the photosynthetic light reaction [82,83]. Interestingly, the microcystin-defect mutant showed decreased glycolate accumulation and an increase in stress metabolites such as trehalose [81]. These results point to role of microcystin in the partitioning of carbon from CBB cycle into photorespiration or alternative routes in microcystin-forming cyanobacteria. Whether the microcystin-binding is somehow influencing RubisCO activity or specificity or if the binding occurs inside the carboxysome and might be reversible is not yet known. Nevertheless, these results show that carbon partitioning may be influenced by complex cellular metabolites, and it will be interesting to analyze if other microcystin-like compounds exert similar functions in other cyanobacteria.

## 3. Regulatory Signals that Cause the Activation or Repression of CCM Genes

### 3.1. Transcriptional Regulation

The structural components of the CCM are encoded by genes that are typically organized as operons, with some being constitutively expressed and others being inducible by exposure to conditions of limited C_i_ availability. A transition from HC to LC conditions results in an up-regulation of transcription of both inducible CO_2_ and HCO_3_^−^ uptake systems [32,71,84,85,86,87,88]. Correspondingly, this leads to large increases in the abundance of the corresponding uptake proteins [28].

As noted, the high affinity HCO_3_^−^ BCT1 transporter is encoded by the *cmpA-D* and was first identified in *Synechococcus* sp. PCC7942 [35]. Subsequently, it was noticed that the orthologous operon in *Synechocystis* sp. PCC6803 is linked to a divergently transcribed gene, *cmpR* [89], that encodes for a transcriptional regulator belonging to the widely distributed protein family of regulators, the LysR transcriptional regulators (LTTR) (Table 1). The LTTRs include both repressors and activators and all known members of this family function through allosteric changes in their DNA binding affinity due to the binding of the small effector molecule [90,91]. Consistent with this mode of operation, it was found that CmpR functions as a transcriptional activator that specifically bound to operator DNA sequences upstream of the RNA polymerase binding and initiation site of the *cmp* operon [89]. Moreover, it was later shown that the binding of CmpR to operator sequences* in vitro* was increased in the presence of RuBP and 2PG providing the first detailed evidence of metabolic signals controlling the transcription of a CCM system [31,92]. During C_i_-limitation, cells are anticipated to accumulate both RuBP and 2PG [59,61] and, thus, these metabolites would be logical effectors of the CmpR activation of the *cmp* operon. According to this model, the accumulation of RuBP and 2PG under low C_i_ conditions would promote the binding of CmpR to its transcriptional activator site leading to the expression of the Cmp bicarbonate uptake system. To test this possibility in intact cells, mutants with changed 2PG accumulation behavior were investigated regarding the expression of the *cmp* operon and other C_i_-regulated genes. In contrast to expectations, the *ccmM* mutant of *Synechocystis* sp. PCC6803, which cannot form carboxysomes and accumulates higher amounts of 2PG even under CO_2_-supplemented conditions, did not show the stronger *cmp* operon expression [61]. However, the increase in 2PG in HC-grown cells was rather low and may be below the threshold for CmpR activation. A complementary attempt was undertaken by overexpressing a 2PG-phosphatase gene in *Synechocystis* sp. PCC 6803, which results in decreased 2PG accumulation in cells shifted from HC into LC conditions. However, it was not accompanied by the expected lower *cmp* operon expression [93]. Again, this unexpected result may reflect that the 2PG accumulation is not exceeding a threshold for allosteric activation of the CmpR. Alternatively, it is possible that the allosteric activation mechanism is more complex than previously believed, requiring, for example, the synergistic binding of the other effector, RuBP, together with 2PG. Such regulatory complexity involving a single transcriptional regulator is not unprecedented. The extensively studied Cbb LTTR sub-family members are known to regulate CBB cycle enzymes in anoxygenic photosynthetic bacteria [94,95]. These LTTRs have complex regulatory interactions that involve allosteric modulation by RuBP, ATP, FBP, and NADPH [94]. Besides these allosteric modulators, some of the Cbb proteins also display protein–protein interactions that modulate their transcriptional activity [95]. Accordingly, it may not be surprising if additional regulatory interactions are discovered for CmpR in addition to its known modulation by RuBP and 2PG [93]. Regulatory complexity may also extend to the gene targets of regulation by a transcriptional regulator. Indeed, CmpR was shown to regulate the transcription of the *psbA* gene, which encodes the photodamage-prone D1 polypeptide of the PSII complex in *Synechococcus* sp. PCC7942 [96]. It is not known whether this “cross-regulation” occurs in other cyanobacteria, but the finding nevertheless illustrates how the regulatory circuits of two adaptive responses, acclimation to light intensity and C_i_ availability, can intersect. In any event, it will be difficult to distinguish among the various possibilities without a more detailed understanding of the* in vitro* kinetic properties of CmpR in relation to its binding to its DNA target as modulated by its allosteric effectors at concentrations that emulate the intracellular environment. As discussed in the previous section, the current metabolomic investigations begin to provide a path in this direction.

**Table 1:** LysR-type regulators in *Synechocystis* sp. PCC 6803.

Gene Name	Synechocystis ORF	Function	Co-regulatory Metabolites	Reference
*ndhR* (*ccmR*)	sll1594	Repressor high affinity C_i_ uptake (genes for CupA, SbtA, Na^+^-NDH-1)	α-KG, NADP^+^	[31,32,89,97]
*cmpR*	sll0030	Activator of ABC-type bicarbonate transporter (*cmp* operon and *psbA* genes)	RuBP, 2PG	[32,35,89,97]
ycf30, *rbcR*	sll0998	Activation of CBB genes	NADPH, 3PGA, RuBP	[89,97,98]
*ntcB*	slr0395	Activation of nitrate assimilation genes	nitrite	[99]

Perhaps, the most critical regulator of the CCM is another LTTR termed NdhR (aka CcmR). In contrast to CmpR, NdhR functions as a repressor [32,97]. Genetic deletion of NdhR produced an aberrant constitutive expression in approximately 20 genes as shown in DNA microarray experiments [32]. These included the gene clusters containing *ndhF3*/*ndhD3*/*cupA*/*sll1735* (*ndh-I_3_* operon), which encode for the structural proteins of the high affinity CO_2_-uptake system genes, and the *sbtA/B* genes, encoding the Na^+^/HCO_3_^−^ symporter (see above). In addition, the genes of the* mnh* operon (*slr2006-slr2013)* are also upregulated in the *ndhR* mutant [29]. The latter genes encode for the polypeptides of an MRP-like [30] NDH-type membrane complex that is proposed to power an outward current of Na^+^ and thereby generate additional membrane electrochemical potential for HCO_3_^−^ uptake via the SbtA/B proteins [32]. However, this assignment remains tentative as discussed in the first section. These observations led to the assignment of a regulon, controlled by NdhR, for the high affinity CCM in *Synechocystis* sp. PCC 6803. Indeed, deletion of the gene encoding NdhR is sufficient to result in the de-repression of all the genes for the major high affinity C_i_ transporters in *Synechocystis* sp. PCC 6803 except for those in the *cmp* operon, which are under the positive regulation by CmpR, as mentioned above. Correspondingly, an increased affinity to C_i_ has been observed in the *ndhR* mutant of the closely related cyanobacterium *Synechococcus* sp. PCC 7002 [38]. In *Synechococcus* sp. PCC 7002, NdhR acts as a negative regulator for all the known CO_2_ responsive genes including the *ndh-I_3_*, *sbt*, and *bic* genes. While the action of NdhR is sufficient to explain the repression of the high affinity CCM under the C_i_ availability downshift conditions [32], additional regulatory mechanisms exist since a protein in the AbrB family of transcriptional regulators, cyAbrB2, also functions as a repressor of the expression of NDH-I_3_ and SbtA/B, at least under certain conditions [100]. Furthermore, since LysR regulators can act as either positive or negative regulators, depending upon where in the promoter region they bind, the microarray experiments may have missed any positive regulation that might be exerted by NdhR with respect to other genes including the ncRNAs that may have regulatory functions as discussed below.

As with CmpR, control of the high affinity C_i_ transport by NdhR is expected to be regulated by small molecules that change in concentration according to the availability of C_i_. Physiological experiments exploring metabolic signals controlling the expression of the high affinity CCM revealed that the putative signaling molecules change in proportion to the size of the intracellular C_i_ pool and are also affected by oxygen tension. The later observation is consistent with the earlier finding that the lag period during the transition from the low affinity to high affinity state depended upon the concentration of O_2_, with the fast mobilization of the high affinity system occurring at ambient conditions (21% *v*/*v*) O_2_ [101]. Using surface plasmon resonance to study the interaction of NdhR with its cognate DNA binding regions of the NdhR regulon, it was shown that NADP^+^ and αKG act as co-repressors through their allosteric interactions with NdhR [31]. In principle, intracellular concentrations of NADP^+^ and αKG are expected to decrease as photosynthesizing cells become starved of C_i_. The decline of NADP^+^ is explained by the continuous action of the light reactions to reduce NADP^+^, while its regeneration due to the consumption of NADPH_2_ by the CBB cycle is slowed due to lack of substrate. Similarly, as carbon fixation by CBB cycle decreases, the flow of carbon into the cyanobacterial TCA cycle may also be expected to decrease, leading to a decrease in the concentration of αKG. However, metabolomic experiments convincingly show that the transition αKG levels remained mainly unchanged at the 3 h time point upon transfer from HC to LC conditions even though 2PG levels were observed to be greatly increased at the same time point [61,72]. As noted, 2PG is a co-activator the BCT-1 expression through its allosteric interaction with the other LTTR, CmpR. Therefore, the unchanged level of αKG at the 3 h time point is a surprise as the NdhR regulon has been upregulated according to gene expression experiments [32,38,88]. A marked drop in the αKG amounts were only observed after 24 h at LC conditions. Probably, the retarded decline in the αKG amounts can be explained by anaplerotic reactions consuming stored glycogen in LC-shifted cells to refill the soluble carbon pools. Accordingly, it would be expected that the high affinity CCM transcript would be repressed under these conditions due to the elevated αKG levels. It is not clear how to reconcile these apparently conflicting data, but at least three explanatory possibilities exist: first, it is possible that other regulatory signals override the co-repressor activity of αKG. Given the multifactorial complexity of the CbbR regulatory interactions in anoxygenic photosynthetic bacteria noted above, it is perhaps likely that NdhR has additional regulatory inputs besides αKG and NADP^+^ and one the additional inputs prevents αKG from exerting its co-repressor activity on NdhR. The second possibility is that the immediate NdhR de-repression is mostly due to an immediate drop in NADP^+^, while the αKG levels are regulating the steady levels in long-term LC-shifted cells. Third, it is possible that the αKG levels only transiently decrease (before the 3 h time frame of the metabolomic experiment sampling time) and this is enough to trigger the accumulation of what would have to be long-lived transcripts for the high affinity CCM.

Given the remaining uncertainties regarding the putative role of αKG in controlling the expression the CCM, it is worth considering an interesting parallel involving tightly regulated glutamine synthase (GS), which is the first committed step in the ammonium assimilation pathway [102]. Here again, αKG figures as a key metabolic effector modulating transcription factor activity. This N-uptake control system is also important also given the well-established observation that C and N assimilation is tightly coordinated to ensure a balanced acquisition of these macronutrients. It has been shown that a decline in αKG rapidly de-represses glutamine synthase inactivating factor (GIF) via NtcA (CRP family of bacterial DNA binding protein) [103]. The rapid de-repression of the *gif* genes due to lower αKG concentrations leads to the rapid inactivation of GS and this can only be reversed by the proteolysis of GIF occurring upon the reestablishment of N-assimilation conditions [104]. Because αKG represents the source of carbon skeletons for ammonium assimilation, the addition of ammonium suddenly depresses the cellular concentration of αKG causing the *gif* genes to be de-repressed and inactivation of GS [73]. With the inhibition GS by the Gif proteins, a major pathway for the consumption of αKG was closed, which soon led to the restoration of αKG levels [73]. Accordingly, the decrease in the αKG level was observed to be a transient event occurring within approximately 30 min. By comparison, microarray experiments analyzing global transcription during the C_i_ downshift showed that the *gif* genes exhibited very fast up-regulation [32] consistent with αKG levels also undergoing a rapid, and possibly transient, decrease in concentration. In addition, the HC to LC shift also markedly decreases the N-assimilation. Thus, it might be the case that after the initial decline in αKG, the amount oscillates to higher levels because of the almost complete stop of net N assimilation and the increased consumption of glycogen. Only, after long-term acclimation the lowered CBB cycle activity and the restart in N-assimilation ensure the stably lowered pool size of αKG. Clearly, experiments to estimate the αKG levels in shorter time points after LC shifts and the analysis of more cyanobacterial strains are necessary to solve this apparent difference in the* in vitro* NdhR regulation model and* in vivo* metabolite and gene expression data. Another set of data, not accounted for in our present understanding of the regulation of the CCM genes, is the observation that down regulation of the 2PG level by overexpression of a 2PG phosphatase significantly depressed the expression of *sbtA* and *ndhF3* [93], controlled by NdhR. However, the latter does not bind 2PG [31] suggesting the involvement of additional, yet unidentified, components. Clearly, additional work needs to be done on the regulatory mechanisms modulating the activity of currently identified transcription factors as well as the distinct possibility that new transcription factors or other players such as small RNAs remain to be discovered.

There is one additional identified member of the LTTR family that is performing an important function in C_i_ metabolism in cyanobacteria: RbcR. This protein is alternatively named CbbR in the annotation of some cyanobacterial genomes because of sequence similarities to the widely distributed LTTR that controls the expression of the enzymes of the CBB cycle in many members of the α-proteobacteria ([95] and citations within). CbbR in *Rhodobacter* spp. controls two major operons containing the genes for RubisCO and other enzymes of the CBB cycle [105]. RbcR in cyanobacteria is also very closely related to an LTTR, termed YCF30, that is found in the plastid genomes of glaucophytes, red algae, and affiliated algae [106]. A recent study of the red algal YCF30 demonstrated that it functions as activator of the transcription of the *rbcLS-cbbX* gene cluster [98]. These genes encode the large and small subunits of RubisCO plus the associated assembly factor, CbbX. Furthermore, the activation of transcription is stimulated by RuBP, 3PGA, and NADPH, which allosterically enhance the binding of YCF30 to its binding site upstream of the *rbcLS-cbbX* gene cluster [98]. On the other hand, little is known about RbcR in cyanobacteria, other than it is essential for viability, based on the observation that it is impossible to entirely delete the gene [89,97]. Nevertheless, essentiality of RbcR is consistent with its assignment as an activator for the similarly configured *rbcLS-cbbX* gene cluster, also found in cyanobacteria. However, unlike the regulation of the CCM genes by CmpR and NdhR, which elicit very large modulations of the corresponding transcript levels, the fold changes in *rbcLS-cbbX* transcripts are quite modest in response to transitions between LC and HC conditions. For completeness, it is important to note that one critical cyanobacterial LTTR, NtcB, also is present in cyanobacteria, although it is involved in nitrogen assimilation. Like the other LTTR, NtcB is allosterically modified by a small molecule, nitrite [99].

In addition to responding to different CO_2_ levels, the transcript abundance of CCM genes also follows changes in the light intensity [25,107]. It is not yet known if these changes also involve the action of NdhR or any other known transcriptional regulator. The two drivers are intimately associated since, in many aspects, the response to a declining CO_2_ supply (reduced sink for electrons) is similar to a rising illumination. Nevertheless, there is a large variability in the reported data to which extent high light influences CCM-related genes. These variations are probably mostly due to large differences between the exact CO_2_ levels used, the history of the cells, and the experimental protocol. In most cases, the CO_2_ level varies between a high (HC) to a low (LC) CO_2_ concentrations (above 1% CO_2_ in air or air level of CO_2_ or lower, respectively). We are missing experiments where a gradual change of CO_2_ level is being imposed to simulate natural conditions that may occur in water bodies or other habitats where cyanobacteria flourish. In addition to variance in the range of physical growth conditions, cyanobacteria also vary substantially in their ability to consume organic carbon from their surroundings. Many strains are obligate photoautotrophs where the sole carbon source is CO_2_, while others are able to perform photomixotrophic or even heterotrophic growth using a wide variety of organic substances [108,109]. Despite its importance to our understanding of cyanobacterial metabolism, little is known about the mechanisms involved in the shifts between photoautotrophic, heterotrophic and photomixotrophic modes of growth, and their regulation. Transcriptional control clearly plays an important role in the regulation of primary carbon and glycogen metabolism under different C- and N-regimes. The alternative sigma factor SigE and the response regulator Rre37 (coded by *sll1330*) have been identified as key actors in the transcriptional regulation of genes for glycogen catabolism via OPP and glycolysis [110,111]. However, it is largely unknown, which signals trigger these transcriptional regulators. Moreover, it will be interesting to know whether or not these proteins somehow are involved in the transcriptional network of NdhR, CmpR and cyAbrB2. It was originally thought, based on phosphorylation patterns [112], that from the point of view of the CCM, photomixotrophic conditions are identical to HC but this is not the case. In *Synechocystis* PCC 6803, low-CO_2_ induced genes are upregulated in the presence of glucose, and glucose-sensitive mutants are far more affected by its presence under HC than in LC [113,114].

In many organisms, soluble adenylate cyclases (SCAs) are supposed to be involved in C_i_ signaling and/or pH regulation. Mammalian sCAs were found to be clearly regulated in the activity by bicarbonate, and a similar regulation was shown for the structurally related enzyme from *Spirulina platensis* [115]. Later on, the regulation of sCA activity by bicarbonate has been also shown for the CyaB1 (Alr2266) from *Anabaena* sp. PCC 7120 [116] and Cya1 (Slr1991) *Synechocystis* sp. PCC 6803 [117]. The latter study revealed that rather CO_2_ than HCO_3_^−^ is influencing the activity of the cyanobacterial sCA enzymes. However, the role of these enzymes in the cyanobacterial HC to LC acclimation is not known despite its regulation via C_i_. The inactivation of *slr1991* resulted in a non-motile mutant of *Synechocystis* sp. PCC 6803 [118]. Unfortunately, the acclimation to LC of the *slr1991* mutant and the possible involvement of sACs and/or AMP have not been investigated yet. Such studies will reveal, whether or not cAMP could represent another C_i_-sensing molecule in addition to the above-discussed metabolites. On the other hand, a potential role for C_i_ in modulating the activity of the cyanobacterial sCA will need to accommodate the kinetic findings showing the concentrations of C_i_ necessary to drive the system are quite high and the above-mentioned indications that the activating species is actually CO_2_ rather than bicarbonate, as initially supposed.

### 3.2. Post-Transcriptional Regulation of the CCM

In addition to the transcriptional control exerted by “classical” transcriptional factors, many hints point at an important role for post-transcriptional regulation of the CCM. It has been shown that only one aspect of the CCM is transcriptionally regulated,* i.e.*, C_i_ uptake as was mentioned before, whereas the increase in carboxysome number in LC-cultivated cyanobacteria is not accompanied by a marked increase in the corresponding transcripts (e.g., [75]). Thus, yet unknown regulatory mechanisms can be expected to participate in the proper acclimation to LC conditions. A new level of regulation was recently found by the action of multiple small RNAs that can act as antisense (as) RNA or as regulatory sRNAs [119]. These RNAs are able to recognize cognate mRNAs via complementary sequences. The association of the mRNA and small regulatory RNA usually decrease the expression of the corresponding genes, because the double-stranded mRNA could be less well recognized by the ribosomes leading to decreased protein synthesis or the RNA hybrid is recognized by RNases leading to an increased RNA turnover. However, there are also some examples that small regulatory RNAs might also have positive effects on mRNA stability or translation. Using the model cyanobacteria *Synechocystis* sp. PCC 6803 and *Anabaena* sp. PCC 7120, transcriptional starting points for the total RNA population were mapped [120,121]. These studies revealed that almost the same number of mRNAs and non-protein-coding small RNAs are encoded by the cyanobacterial genomes, whereby some of the small RNAs reached very high abundances comparable to the highest expressed mRNAs from protein-coding genes. Interestingly, a high dynamic of transcriptional start sites was found when *Synechocystis* sp. PCC 6803 was cultivated under 10 different environmental conditions, leading to the identification of more than 5000 active promoters [122]. The authors also compared gene expression in cells grown at different CO_2_ conditions. Among the multiple changes, one possible regulatory sRNA was detected that specifically responded to different CO_2_ conditions,* i.e.*, it was always detected in cells grown at LC regardless of the other stress conditions, but disappeared specifically when the cells were exposed to HC. Thus, the sRNA was named CsiR1, carbon stress-induced RNA1 [122]. It will be interesting to know, which genes are targeted by CsiR1 and which role it plays in the LC acclimation. The functions of only a few small RNAs are known so far. For example, one asRNA was found to interact specifically with the mRNA for the flavodiiron protein Flv4 [123]. This protein is highly induced in cells shifted from HC to LC conditions and thought to be involved in the protection of photosystem II. It was shown that the asRNA AS1_flv4 really binds to the *flv4* mRNA and prevents the immature expression of the *flv4-2* operon. Moreover, the LC-induced expression of AS1_flv4 was found to be mediated via the action of cyAbrB2 [123], a transcriptional factor somehow involved in the regulation of many LC-regulated genes [100]. Recently, with PsrR1 the first small regulatory RNA was identified that regulates many mRNA targets mostly coding for proteins of photosystem I subunits [124]. Among the possible target RNAs, the authors reported that of one *ccmK* gene, thus this sRNA might not only regulate photosynthesis genes but also some CCM genes according to the light regime. An overlap between the high light response and the acclimation to LC has been already recognized in microarray experiments analyzing mRNA expression in *Synechocystis* sp. PCC 6803 [25,107]. Regardless of whether the details of the small regulatory RNA control of gene expression mechanisms are determined, the general point that can be taken is that the regulation of the CCM occurs at multiple levels. As shown in Figure 2, the acclimation of cyanobacteria to changing levels of external C_i_ involves a network of interactions where the intracellular concentrations of specific “regulatory metabolites” respond to the availability of C_i_ and act to allosterically modify the activity of transcriptional regulators. Gene expression is further tuned by the action small regulatory RNAs that function to control the levels and/or translation of the mRNAs encoding the CCM.

## 4. Conclusions

The general function, structure and regulation of the cyanobacterial CCM has been analyzed in great detail during recent years. Almost all proteins and the corresponding genes for CCM components are known and well characterized in model cyanobacteria. The increasing number of complete genome sequences from cyanobacteria of different ecological and phylogenetic origins now allows investigating, whether or not distinct differences in the function and structure of the CCM evolved as adaptation to different ecological niches. Moreover, the timing of the primary origin of the CCM is still not known and should be analyzed using the now existing huge genome databases. Many of the genes coding for CCM components show differential regulation as a response to different CO_2_ concentrations but also to many other environmental stimuli such as high light or the availability of organic carbon sources. Especially, the LC-induced up-regulation of the C_i_ transporters via the action of LysR-type transcriptional factors is quite well understood in some model strains and in biochemical studies. However, even in these cases, the* in vivo* integration of metabolic signals, transcriptional factor activities and gene expression kinetics shows some conflicting data pointing at a more complex regulatory network in the living cells than anticipated from the analysis of isolated components* in vitro*. Compared to regulation of the C_i_ uptake components, mechanisms guaranteeing the increase of carboxysome number and the rerouting of the carbon assimilation are almost unknown. Here, novel players seem to be involved that act independently from the transcriptional factors. Small regulatory RNAs and direct posttranslational regulation of protein activities are prime candidates for additional levels of C_i_-depending acclimation processes.
